# Methotrexate and relative risk of dementia amongst patients with rheumatoid arthritis: a multi-national multi-database case-control study

**DOI:** 10.1186/s13195-020-00606-5

**Published:** 2020-04-06

**Authors:** Danielle Newby, Daniel Prieto-Alhambra, Talita Duarte-Salles, David Ansell, Lars Pedersen, Johan van der Lei, Mees Mosseveld, Peter Rijnbeek, Glen James, Myriam Alexander, Peter Egger, Jana Podhorna, Robert Stewart, Gayan Perera, Paul Avillach, Solène Grosdidier, Simon Lovestone, Alejo J. Nevado-Holgado

**Affiliations:** 1 Department of Psychiatry, Warneford Hospital, University of Oxford, Oxford, UK; 2 Department of Biomedical Informatics, Harvard Medical School, Boston, USA; 3 Centre for Statistics in Medicine, NDORMS, University of Oxford, Oxford, UK; 4 Fundació Institut Universitari per a la recerca a l’Atenció Primària de Salut Jordi Gol i Gurina (IDIAPJGol), Barcelona, Spain; 5 Quintile IMS, London, UK; 6 Department of Clinical Epidemiology, Aarhus University Hospital, Aarhus, Denmark; 7 Department of Medical Informatics, Erasmus Universitair Medisch Centrum, Rotterdam, Netherlands; 8 Real World Data, GlaxoSmithKline, Uxbridge, UK; 9 Boehringer Ingelheim International GmbH, Medicine CNS & Emerging Areas, Ingelheim am Rhein, Germany; 10 Institute of Psychiatry, Psychology and Neuroscience, Kings College London, London, UK; 11 South London and Maudsley NHS Foundation Trust, London, UK; 12 Janssen-Cilag’, Beerse, Belgium

**Keywords:** Dementia, Rheumatoid arthritis, Inflammation, Anti-inflammatory drugs, DMARDs, Methotrexate, Sulfasalazine, Case-control study, European Medical Information Framework, EMIF

## Abstract

**Background:**

Inflammatory processes have been shown to play a role in dementia. To understand this role, we selected two anti-inflammatory drugs (methotrexate and sulfasalazine) to study their association with dementia risk.

**Methods:**

A retrospective matched case-control study of patients over 50 with rheumatoid arthritis (486 dementia cases and 641 controls) who were identified from electronic health records in the UK, Spain, Denmark and the Netherlands. Conditional logistic regression models were fitted to estimate the risk of dementia.

**Results:**

Prior methotrexate use was associated with a lower risk of dementia (OR 0.71, 95% CI 0.52–0.98). Furthermore, methotrexate use with therapy longer than 4 years had the lowest risk of dementia (odds ratio 0.37, 95% CI 0.17–0.79). Sulfasalazine use was not associated with dementia (odds ratio 0.88, 95% CI 0.57–1.37).

**Conclusions:**

Further studies are still required to clarify the relationship between prior methotrexate use and duration as well as biological treatments with dementia risk.

## Background

Dementia is one of the largest unmet medical needs, and with growing numbers of older people in a majority of countries worldwide, it is set to become an increasing burden on health services and economies [1]. Despite considerable progress in the understanding of the pathological lesions associated with the diseases causing the commonest forms of dementia—Alzheimer’s disease (AD), vascular dementia, dementia with Lewy bodies and fronto-temporal dementia—this has not resulted in therapeutic progress, with multiple trials of compounds designed for disease modification failing, often at a great cost [2]. Whilst part of the failure of such trials might be that they have largely been conducted in patients with established disease, possibly too late for therapeutic benefit, there is an increasing realisation that it is likely forms of dementia are part of a complex set of pathological processes [3]. Understanding these causal pathways, and in particular, understanding their timing in relation to the onset of dementia, has become a pressing task in order to progress a novel approach to therapeutic development.

One of the processes that has come under intensive investigation in recent years has been that of inflammation [4–6]. In the analysis of large-scale genome-wide association studies, inflammatory pathways such as complement signalling were consistently identified as altering susceptibility to disease [7, 8]. Post-mortem studies reflect this association with inflammation, as microglial and astrocyte numbers are increased and associated with pathological lesions in disease [9]. However, such genetic and pathological studies are unable to distinguish between cause and effect, and it might be that an inflammatory reaction is part of the defence mechanism or is contributing to disease progression [10].

An alternative approach to understanding the direction and timing of the effect of inflammation in relation to dementia is to utilise existing observational clinical data where individuals have received anti-inflammatory drugs. It was such an approach that first highlighted the role of inflammation in neurodegeneration when it became apparent that non-steroidal anti-inflammatory compounds were associated with reduced dementia risk [11–14].

Many subsequent studies have reproduced this finding, although trials of such compounds have not been successful to date, perhaps because the association is only seen in those taking medication for extended periods of time before the onset of dementia [15]. In line with this, we recently found non-steroidal anti-inflammatory drugs (NSAIDs) were associated with higher cognitive function in participants in the UK Biobank cohort study of largely healthy individuals [16]. Rather than being cognitive enhancers, a potential explanation of this finding is that NSAIDs are reducing the decline in cognitive function in pre-clinical dementia states. Together, these real-world observational clinical data studies, therefore, have made a significant contribution in the following: first, adding support to the association with inflammation in disease; second, in adding support suggesting a direction of that effect; and third, in providing evidence for timing in relation to disease and therefore informing the design of clinical studies.

In recent years, more specific anti-inflammatory drugs have become part of the pharmacotherapy of disorders such as rheumatoid arthritis, providing an opportunity to begin to explore the effects of different mechanisms of inflammation in relation to neurodegeneration [17, 18]. Recognising this, we recently used Clinical Practice Research Datalink (CPRD), a large dataset derived from primary care in the UK, to explore the relationship between different anti-inflammatory compounds and showed that disease-modifying antirheumatic drugs (DMARDs), and especially methotrexate, are associated with a reduction in risk of incident AD [19]. However, although a relatively large study, the data was from a single source and in a single health care context and thus vulnerable to unanticipated confounding. Here, we set out to replicate and extend this study across multiple real-world observational datasets across Europe.

## Methods

### Study design, setting and source of data

We used a nested retrospective case-control study design to investigate the association between the use of disease-modifying antirheumatic drugs (methotrexate and sulfasalazine) and the incidence of dementia diagnosis in routine healthcare data. We used electronic health records (EHRs) available via the European Medical Information Framework (EMIF; www.emif.eu), a public-private partnership under the EU Innovative Medicines Initiative [20]. Methotrexate and sulfasalazine were selected as follows: (1) both drugs are first-line treatments for rheumatoid arthritis, (2) there is no universally accepted prescribing preference for these drugs when clinicians are faced with the same symptoms and (3) each drug has a different mechanism of anti-inflammatory action [21, 22].

We selected the four databases with primary care, hospital and pharmacy dispensing data sources from across Europe including Integrated Primary Care Information (IPCI, 1.8 million) [23], Aarhus University Prescription Database (AUH, 1.8 million) [24], The Health Information Network (THIN, 3.8 million) [25, 26] and Information System for Research in Primary Care (SIDIAP, 5.5 million) [27, 28]. Data collection in IPCI, AUH, THIN and SIDIAP was initiated in 1995, 2000, 2002 and 2006, respectively. Data collection for this study was from the first initiation of each database to 2016. Further information regarding the databases utilised is provided in the supplementary information (Table S1).

### Participants and study size

The case population is formed by those individuals who were identified with a first-ever clinical or referral record of dementia. Further information regarding dementia definitions and mapping to the different databases in this study can be found in Perera et al. [29]. The clinical codes for dementia and other disease variables can be found in the supporting information (Table S2).

**Fig. 1:**
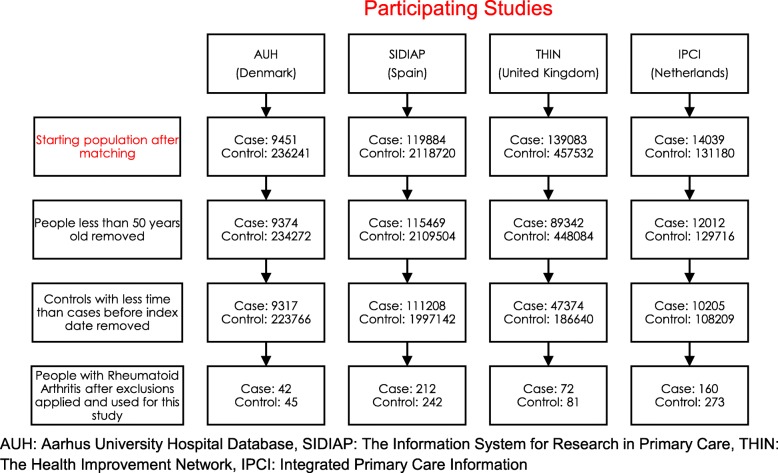
The number of individuals initially included in the study and the number of individuals utilised for analysis after exclusions

The date at which a patient received the first diagnosis of dementia was defined as the index date. Up to 25 controls from the initial study population per case were matched on age (± 2 years), gender and GP practice. Up to 25 controls were utilised as it was anticipated that when only including those people with a previous history of RA for analysis, the number of controls would decrease. For the AUH database, information on GP practice was not available; therefore, controls were matched to cases on age and gender only. All individuals included in the analysis had been registered in their respective database for at least 1 year before the index date. The index date for controls (with no dementia diagnosis at their index date or before) was chosen as the date of dementia diagnosis of their matched dementia case. From this initial population of cases and controls, only those people who were 50 years or older at the index date were included in the study. Furthermore, for each case, matched controls who had a shorter period before the index date were removed. Finally, only those people with a diagnosis of rheumatoid arthritis (RA) before the index date were included in this study. Any cases with no remaining controls after applying exclusion criteria were excluded. A population flowchart showing the individual numbers of cases and controls per dataset is shown in Fig. 1.

### Outcome and exposures

The outcome variable was defined as the presence or absence of a diagnosis of dementia as a binary variable. The diagnosis of dementia was identified in each database as a first-ever clinical or referral record of dementia from 1 January 1995 to 31 December 2016. The diagnosis of rheumatoid arthritis was identified in each database as a first-ever clinical or referral record of rheumatoid arthritis. The exposure variables in this study were as follows: first prescription of methotrexate, if any; first prescription of sulfasalazine, if any; number of days using methotrexate before index date; and number of days using sulfasalazine before the index date. The number of days of drug use was calculated from the first date of drug use to the last date of drug use followed by a 12-month gap all before the index date. Days of use for these drugs were converted to years by dividing by 365.25 for consistency. In order to standardise drug treatments across the different databases if information on dosing is available, the duration was calculated by dividing the amount of drug prescribed and the actual dosing of the individual patient. If information on dosing was lacking or in case of missing dose, the total amount (per prescription) is divided by the recommended dosing according to the Summary of Product Characteristics (SmPC)/defined daily dose of the respective drug. ATC drug codes for the two anti-inflammatory medications were L01BA01 and L04AX03 for methotrexate and A07EC01 for sulfasalazine. All databases utilised ATC codes for methotrexate and sulfasalazine apart from THIN, which utilises ATC and Read coding.

### Potential confounders and model adjustments

Factors considered potential confounders were identified including age at index date, highest body mass index measurement (BMI) 2–12 years before the index date (either BMI or calculated from height and weight measurements) and comorbidities at or before the index date (stroke and acute myocardial infarction (AMI)). Age was included as a confounder in all models due to the 2-year time window used for matching. The highest BMI 2–12 years from the index date was used when available, but calculated from weight and highest height recorded in adulthood otherwise. The highest BMI was utilised as dementia patients are more likely to lose weight leading up to diagnosis [30]. Any BMI value less than 15 kg/m^2^ or greater than 60 kg/m^2^, any weight measurement less than 30 kg or greater than 150 kg and any height measurement less than 1 m or greater than 2.5 m were considered measurement errors and not considered. Cardiovascular disease, including stroke, has been hypothesised as lying on the causal pathway between RA and dementia, where people with RA are more at risk of developing cardiovascular disease such as stroke, which is then a risk factor for dementia [31, 32]; therefore, models were used with and without this previous history of stroke.

We initially performed an unadjusted model and then we adjusted for age at the index date (years), diagnosis of stroke before the index date (yes or no), diagnosis of AMI (yes or no) and BMI, and finally, fully adjusted models with all confounders but without the previous stroke diagnosis were performed.

### Missing data

The only confounding variable with missing data was the highest BMI (2–12 years) with missingness of 39.57%. BMI was imputed to avoid the exclusion of patients and to reduce selection bias. An imputed dataset was generated using 10 multiple imputations by chained equations to replace these missing values. We included all confounders, diagnosis of hypertension, dementia diagnosis and both drug exposures in the imputation process.

### Statistical methods

A descriptive summary table of the dataset utilised is presented in Table 1. For the descriptive summary statistics used in this study, an unpaired *t* test was applied for normally distributed numerical demographics, a Wilcoxon unpaired *t* test was applied for non-normally distributed numerical demographics and a chi-square test for categorical demographics was applied.

**Table 1:** Characteristics of cases and controls (with and without dementia) with rheumatoid arthritis used in this study

	Case (with dementia diagnosis)	Control (no dementia diagnosis)	*p* value
*n*	486	641	–
Age at index date (years)	79.8 (± 6.89)	79.5 (± 7.02)	0.522
Gender	86.4% F	87.4% F	0.707
Time since rheumatoid arthritis diagnosis before the index date (years)	6.87 (± 6.29)	7.06 (± 5.92)	0.232
Prescribed with sulfasalazine	40 (8.23%)	57 (8.89%)	0.776
Years of sulfasalazine use before the index date	2.72 (± 3.57)	2.57 (± 3.96)	0.457
Prescribed with methotrexate	123 (25.3%)	178 (27.77%)	0.392
Years of methotrexate use before the index date	2.69 (± 3.06)	3.80 (± 2.44)	0.002
Prescribed with methotrexate or sulfasalazine	19 (3.91%)	25 (3.90%)	1.000
Diagnosed with AMI	26 (5.35%)	34 (5.30%)	1.000
Diagnosed with stroke	37 (7.60%)	64 (10.00%)	0.202
Highest BMI before the index date	28.1 (± 4.99)	28.8 (± 4.90)	0.061
BMI missingness	178 (36.62%)	211 (32.92%)	0.217
Total time in database (years)	7.64 (± 7.82)	7.77 (± 7.66)	0.301
Time in database before the index date (years)	5.16 (± 7.47)	4.62 (± 7.01)	0.027

Case, diagnosis of dementia; control, no diagnosis of dementia. For the calculation of *p* values to compare the demographics between cases and controls, an unpaired *t* test was applied for normally distributed numerical demographics, a Wilcoxon unpaired *t* test was applied for non-normally distributed numerical demographics and a chi-square test for categorical demographics was applied

Conditional logistic regression was used to estimate the association between drug use and dementia diagnosis using R version 3.4.3 and the “survival” package [33]. The data from each database was combined into one dataset, and a variable indicating which database the data had originated from was created. We first calculated the odds ratios (ORs) with 95% confidence intervals (CI) from crude models with no adjustment (model 0). Secondly, we adjusted for all confounders listed in the “Potential confounders and model adjustments” section: age at index date (years), diagnosis of stroke before the index date (yes or no), diagnosis of AMI (yes or no) (model 1) and database (multi-level variable). Finally, we calculated the models with all confounders but without the previous stroke diagnosis (model 2).

To test the impact of days of use of anti-inflammatory medications before dementia diagnosis, a new variable was created based on the days of use of either methotrexate or sulfasalazine. For each medication, days of use before the index date were converted into tertiles for those taking medication. This created a multi-level variable of the different tertiles based on the days of use of the medication. Those not taking the medication were as the reference level in the analysis. The four groups created for methotrexate days of use variable were as follows: group A (reference level *n* = 740), not taking methotrexate; group B (*n* = 52), more than 0 years but less than or equal to 1.18 years (432.33 days) of methotrexate use before the index date; group C (*n* = 51), those with greater than 1.18 years but less than or equal to 4.11 years (1500.8 days) of use of methotrexate before the index date; and finally, group D (*n* = 51), those taking methotrexate for more than 4.11 years. The four groups created for sulfasalazine days of use variable were as follows: group A (reference level), not taking sulfasalazine; group B, more than 0 days but less than or equal to 0.49 years (177.5 days) of sulfasalazine use before the index date; group C, those with greater than 0.49 years but less than or equal to 2.21 years (808 days) of use of sulfasalazine before the index date; and finally, group D, those taking sulfasalazine for more than 2.21 years. This multi-level variable, containing the different tertiles based on days of use for either methotrexate or sulfasalazine, was then included into the models (instead of the binary variable indicated if the drug was taken by the patient), and models 0–2 were calculated using this multi-level variable.

## Results

### Descriptive data

A total of 486 subjects with RA who developed dementia (cases) and 641 matched controls also with RA were included. A study participant flowchart is provided showing the impact of each exclusion criterion (Fig. 1). Characteristics of individuals included in the study are shown in Table 1. IPCI had 433 patients (37% cases), SIDIAP 454 (47% cases), THIN 153 (47% cases) and AUH 87 (48% cases).

Cases and controls were balanced in terms of most of the measured variables (Table 1). Additionally, although the total time in the database between cases and controls did not differ, controls had slightly shorter time in the databases compared with the cases with time in the database before the index date at 5.16 years for cases and 4.62 years for controls. However, this difference is not present in three databases (AUH, SIDIAP and THIN) when considering the individual databases. The difference is due to the IPCI dataset having more cases with a higher number of controls who have a shorter time before the index date. Other covariates did not differ between cases and controls to a statistically significant extent. The total range of ages at the index date for cases and controls in Table 1 was 50–97 years and 52–97 years, respectively. The total range of methotrexate use in years was 0.074–18.8 years, and the total range of sulfasalazine use in years was 0.036–19.5 years. For a detailed breakdown of the duration of methotrexate and sulfasalazine use across 5-year age brackets for dementia cases, see the supporting information (Table S3).

### Association between DMARDs use and dementia diagnosis

For the unadjusted model, there was a trend, although not statistically significant, towards reduced risk of dementia with methotrexate use (OR 0.76, 95% CI 0.56–1.03). Using adjusted models, we found a statistically significant association with methotrexate but no association with sulfasalazine in relation to dementia diagnosis. For models 1 and 2, similar ORs were obtained for methotrexate (0.71, 95% CI 0.52–0.98; 0.71, 95% CI 0.52–0.98) both achieving statistical significance (Table 2). As models 1 and 2 show similar results, we conclude that there is no significant effect of stroke on the relationship between drug exposure and dementia onset. A forest plot showing the results of Table 2 can be found in the supplementary information (Figure S1).

**Table 2:** Unadjusted and adjusted model showing the association between methotrexate and sulfasalazine treatment and dementia diagnosis in people with rheumatoid arthritis using conditional logistic regression

Drug	Model 0		Model 1		Model 2	
	Unadjusted OR (95% CI)	*p* value	Adjusted OR (95% CI)	*p* value	Adjusted OR (95% CI)	*p* value
Methotrexate	0.76 (0.56–1.03)	0.076	0.72 (0.52–0.98)	0.036	0.71 (0.52–0.98)	0.034
Sulfasalazine	0.88 (0.57–1.36)	0.56	0.89 (0.57–1.38)	0.60	0.88 (0.57–1.37)	0.57

Model 0, no adjustment; model 1, adjustment for age, BMI, stroke and AMI; model 2, same as model 1 but not including diagnosis of stroke. *OR* odds ratio for dementia risk

**Fig. 2:**
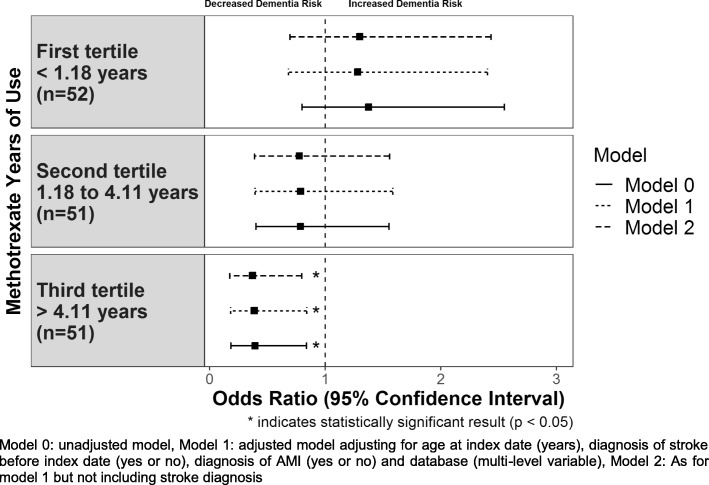
Association between methotrexate years of use and dementia diagnosis in people with rheumatoid arthritis using conditional logistic regression

We then investigated the number of days of use of methotrexate and sulfasalazine before dementia diagnosis. The results showing the number of days of use of methotrexate in tertiles with dementia diagnosis are shown in Fig. 2.

We found a clear treatment time effect (even though tertiles 1 and 2 were not significant) with those taking methotrexate for the longest period (greater than 4.11 years) prior to the index date having the greatest effect on dementia risk for all models (Fig. 2). The results indicate that the association is only significant for those who took methotrexate for a minimum of over 4 years of treatment. For the unadjusted model, those who took methotrexate for a minimum of 4.11 years achieved an odds ratio of 0.39 (95% CI 0.18–0.84, *p* value 0.015) with similar odds ratios obtained for models 1 and 2 of 0.38 (95% CI 0.18–0.84, *p* value 0.016) and 0.37 (95% CI 0.17–0.79, *p* value 0.011), respectively.

The full table of results can be found in the supplementary information (Table S4). We then repeated this analysis for sulfasalazine finding no significant association between duration of prescription and risk of dementia (Table 3). However, it is interesting to note that the general trend (even though all values are not significant) indicating longer use is associated with increased dementia risk.

**Table 3:** Unadjusted and adjusted model showing the association between days of use of sulfasalazine treatment and dementia diagnosis in people with rheumatoid arthritis using conditional logistic regression

Variable group	Model 0		Model 1		Model 2	
	Unadjusted OR (95% CI)	*p* value	Adjusted OR (95% CI)	*p* value	Adjusted OR (95% CI)	*p* value
No sulfasalazine use	Ref		Ref		Ref	
Sulfasalazine use for 0.49 years (first tertile)	0.60 (0.28–1.31)	0.20	0.65 (0.30–1.41)	0.28	0.65 (0.30–1.41)	0.28
Sulfasalazine use for 0.49 to 2.21 years (second tertile)	0.93 (0.45–1.93)	0.85	0.94 (0.45–1.95)	0.86	0.89 (0.43–1.85)	0.76
Sulfasalazine use for > 2.21 years (third tertile)	1.21 (0.58–2.51)	0.62	1.16 (0.55–2.44)	0.69	1.19 (0.57–2.48)	0.65

Model 0, no adjustment; model 1, adjustment for age, BMI, stroke and AMI; model 2, same as model 1 but not including stroke variable. *OR* odds ratio for dementia risk

## Discussion

In this uniquely large and geographically diverse sample using four European harmonised electronic health record datasets using the EMIF platform, we found evidence that in adults older than 50 years of age with rheumatoid arthritis, taking methotrexate was associated with a lower risk of a subsequent dementia diagnosis, specifically recorded methotrexate use for more than 4 years. We also investigated sulfasalazine use but found no such association.

However, the lack of association between sulfasalazine and dementia could also be attributed to the low frequency of sulfasalazine usage in these databases, and this could provide an account for the lack of statistically significant reduction in dementia risk.

The data used in this study does not permit any conclusive causal information regarding the biological mechanisms of the anti-inflammatory properties of methotrexate relative to sulfasalazine in relation to dementia risk. However, by identifying the potential mechanisms and how they differ between the two drugs will be helpful in pointing to future directions for understanding the underlying mechanisms in basic research of inflammation and dementia, which would need to be understood before any such compounds would be tested in intervention trials. The exact mechanism of action of methotrexate or sulfasalazine is not well understood; however, several overlapping and distinct mechanisms have been proposed. For methotrexate, these mechanisms include folate-dependent processes, adenosine signalling, inhibition of methyl-donor production, generation of reactive oxygen species, alteration of cytokine profiles and downregulation of adhesion molecule expression, eicosanoids and matrix metalloproteinases [34]. For sulfasalazine, it is still not clear whether the parent drug and/or its main metabolites (sulfapyridine and mesalazine) are responsible for the beneficial effects in rheumatoid arthritis [35, 36]. Based on in vitro and in vivo studies, sulfasalazine appears to exert effects on inflammatory cell function, cytokine and antibody production, inhibition of folate-dependent enzymes, inhibition of synovial neovascularization and free radical scavenging activity [37]. Both methotrexate and sulfasalazine appear to be involved in adenosine signalling [38–40] and affect T cell activity via CD8 and inhibit B cells by interrupting Ig synthesis whereas only methotrexate interferes with IL-2, and only sulfasalazine causes prostaglandin inhibition. There is evidence in the literature that methotrexate is involved in the inhibition of inflammatory cell proliferation, interference with T cell activity and cytokine secretion, and augmented release of adenosine, macrophage and polymorphonuclear leukocyte inhibition where this has not been shown with sulfasalazine [37, 38]. These drugs are involved in overlapping and distinct mechanisms, and this could potentially explain why we did not see an association between sulfasalazine and dementia risk. However, the lack of cumulative measures reflecting inflammatory disease burden precludes definitive conclusions regarding the causal relationship between methotrexate use, reduced inflammation and lowered risk of dementia.

Investigations of anti-inflammatory DMARDs on dementia risk have reported conflicting results. Judge et al. [19] showed a decreased risk of dementia with methotrexate use. Using the UK Clinical Practice Research Datalink, Judge et al. found that DMARD users were at reduced risk of dementia (hazard ratio 0.60; 95% confidence interval 0.42–0.85), and the effect was strongest in methotrexate users (hazard ratio 0.52; 95% confidence interval 0.34–0.82). Our study extends these findings by utilising datasets across multiple countries as well as highlighting the stronger associations of longer methotrexate use with reduced dementia risk.

Judge et al. contradict the study carried out by Chou et al. [41]. In this study, using the Taiwan National Health Insurance Research Database, people taking non-biologic DMARDs had a higher risk of any dementia type (AD, vascular) compared with people who did not take DMARDs or used biologic DMARDs. This study used a cut-off age of people equal or older than 20 years; however, the majority of people used in the analysis were older than 65 (> 80%). Furthermore, this study also showed that the numbers of days of use of either methotrexate or sulfasalazine increased dementia risk. The contradictory results from this study could be for a number of reasons, firstly differences in geographic locations (European versus Asian). Further studies are still required to clarify the relationship between prior methotrexate use and duration as well as dementia risk with the use of other DMARDs, particularly biological therapies. Future experimental studies are warranted to determine the specific mechanisms for the protective association between prior methotrexate treatment and dementia.

Our study has several strengths. First, the use of multiple routine EHR databases under the EMIF platform has allowed for aggregation and harmonisation of huge volumes of suitable data to investigate the association between dementia and inflammatory exposures. The access to large amounts of data has meant that we have been able to select people with a relatively rare condition such as RA and have sufficient numbers of incident cases of dementia. By only including people who had RA in this study, we have increased the likelihood of cases and controls being comparable rather than just comparing medication use or not within the total samples and therefore could consider the effect of medications and dementia risk. In this study, we used a matched population, which minimises bias due to confounding by indication but also limits the generalizability of the findings. In particular, we only utilised those people with RA also limiting our generalizability. In this study, four EHR databases, primary care and hospital-based, from four European countries, were utilised, giving general applicability across multiple European countries. Second, we followed an EMIF harmonisation process where a single protocol was utilised for the study, single format of data extracts, semantic harmonisation process ensuring comparability of clinical code lists and a statistical analysis plan with the same statistical analysis steps applied on the extracted data ensuring consistency in results.

Our study also had limitations. We only investigated the effect of two anti-inflammatory drugs, methotrexate and sulfasalazine, without considering the impact of other drug treatments potentially administered such as biologics and concomitant use of biologics and other non-biologic anti-inflammatory medications. Therefore, confounding by indication could exist as the severity of RA may impact the choice of medication where methotrexate and sulfasalazine are usually the first-line treatments for RA. Therefore, the characteristics of patients receiving methotrexate versus sulfasalazine could be different due to the underlying disease severity (as measured by the Disease Activity Score 28 (DAS28), presence/absence of joint erosions and other inflammatory biomarkers) and degree of inflammation and therefore could contribute to confounding by indication. However, Judge et al. [19] used propensity score matching to account for confounding by indication and showed similar results to ours regarding a reduced risk of dementia with methotrexate use. Furthermore, drug adherence could affect the results with drug side effects which could affect the compliance of taking the medications utilised in this study. Additionally, discontinuation of drug treatment could impact the degree of undefined inflammation leading up the onset of dementia in cases. Based on the study design, it is feasible that some patients had continuous treatment and others did not. Additionally, cases and controls who used both methotrexate and sulfasalazine were included in the analysis, adding another potential confounder that weakens the overall statistical association between methotrexate use and lowered risk of dementia. Owing to the observational nature of the study, there remains the potential for residual confounding that could attenuate or explain the observed associations, due to unmeasured variables such as arthritis disease severity, health, concomitant therapy and lifestyle effects such as physical activity and alcohol consumption. However, in spite of this, the data used for this study does reflect the wider rheumatoid arthritis patient population in terms of gender distribution [42].

Regarding the clinical diagnosis of dementia, there is the possibility of non-uniform case assignment. However, it is reasonable that all countries (the UK, Spain, Denmark and Holland) have dementia assessment services and the potential for specialist diagnoses (which are more likely to vary in coverage within rather than between sites) and that this will be more likely to have obscured our findings through case heterogeneity and measurement error rather than give rise to false-positive findings. Furthermore, the diagnosis of dementia is usually assessed by a general practitioner and not by a standardised research diagnosis, which may differ depending on the type of dementia, so this may attenuate the associations. We also defined dementia to encompass any type of dementia including Alzheimer’s disease and vascular dementia; therefore, it might be possible that a specific subtype of dementia is driving the association.

Further work, from this study, would be to establish the following: firstly, if other anti-inflammatory medications such as hydroxychloroquine and leflunomide affect dementia risk; secondly, whether biological anti-inflammatories reduce dementia risk; thirdly, if these associations are effected by NSAID and prednisone usage; and finally, whether methotrexate treatment affects the risk in the different dementia subtypes.

## Conclusion

In summary, this case-control study across multiple EHR databases found that prior methotrexate use, but not sulfasalazine use, was associated with dementia diagnosis even after adjusting for patients’ demographics and comorbidities. Furthermore, we showed that there is a gradual decrease against dementia risk the longer the use of methotrexate before the index date. These findings are relevant from a public health perspective since they highlight the potential of methotrexate to protect against dementia; however, the mechanism behind this remains to be elucidated.

## Supplementary information

**Additional file 1 **: Supporting Information.xlsx Description of data: Excel document containing 1). **Table S1.** of characteristics of databases utilised in this study 2). **Table S2.** of clinical diagnosis codes for disease variables 3). **Table S3.** of the age specific breakdown of dementia occurrence in 5 year intervals with duration of drug treatment usage 4). **Table S3.** showing the association between of days of use of methotrexate treatment and dementia diagnosis in people with rheumatoid arthritis and 5). **Figure S1.** showing the association between methotrexate and sulfasalazine treatment with dementia diagnosis in people with rheumatoid arthritis.

## Data Availability

The datasets analysed during the current study are not publicly available due to the confidentiality of patients but are available via application to individual data custodians under the EMIF platform (http://www.emif.eu/).

## Abbreviations

AD: Alzheimer’s disease
MTX: Methotrexate
SUL: Sulfasalazine
DMARDs: Disease-modifying antirheumatic drugs
NSAIDs: Non-steroidal anti-inflammatory drugs
EMIF: European Medical Information Framework
ATC: Anatomical Therapeutic Chemical
THIN: The Health Information Network
AUH: Aarhus University Prescription Database
IPCI: Integrated Primary Care Information
SIDIAP: Information System for Research in Primary Care
ICD-9/10: International Classification of Diseases
ICPC: International Classification of Primary Care
RA: Rheumatoid arthritis
BMI: Body mass index
AMI: Acute myocardial infarction
